# Estimating the Fiscal Effects of Public Pharmaceutical Expenditure Reduction in Greece

**DOI:** 10.3389/fpubh.2015.00203

**Published:** 2015-08-31

**Authors:** Kyriakos Souliotis, Manto Papageorgiou, Anastasia Politi, Nikolaos Frangos, Yiannis Tountas

**Affiliations:** ^1^Faculty of Social and Political Sciences, University of Peloponnese, Corinth, Greece; ^2^Department of Statistics, Athens University of Economics and Business, Athens, Greece; ^3^Medical School, National and Kapodistrian University of Athens, Athens, Greece

**Keywords:** public pharmaceutical expenditure, fiscal consolidation, multipliers, unemployment, Greece, revenue

## Abstract

The purpose of the present study is to estimate the impact of pharmaceutical spending reduction on public revenue, based on data from the national health accounts as well as on reports of Greece’s organizations. The methodology of the analysis is structured in two basic parts. The first part presents the urgency for rapid cutbacks on public pharmaceutical costs due to the financial crisis and provides a conceptual framework for the contribution of the Greek pharmaceutical branch to the country’s economy. In the second part, we perform a quantitative analysis for the estimation of multiplier effects of public pharmaceutical expenditure reduction on main revenue sources, such as taxes and social contributions. We also fit projection models with multipliers as regressands for the evaluation of the efficiency of the particular fiscal measure in the short run. According to the results, nearly half of the gains from the measure’s application is offset by financially equivalent decreases in the government’s revenue, i.e., losses in tax revenues and social security contributions alone, not considering any other direct or indirect costs. The findings of multipliers’ high value and increasing short-term trend imply the measure’s inefficiency henceforward and signal the risk of vicious circles that will provoke the economy’s deprivation of useful resources.

## Introduction

Detecting sources of vulnerability in the economy timely, especially in unstable financial conditions, is a rational act keyed to the design of sound government fiscal policies on the basis of well-reasoned decisions. The process of dissolving uncertainty requires the analytical study of national account data and economic flows and can be strengthened through the use of fiscal statistics tools [see Government Finance Statistics Manual 2001 (1)].

The Greek Government, in order to deal with the dramatic economic fluctuations caused by the global financial crisis (2), proceeded to a great number of fiscal policies in comparison with the other European Union (EU) member states (3). The drastic adjustment of Greece’s economy to the macroeconomic fluctuations and stresses was designed with a view to cutting public spending, rather than raising revenues (4, 5) and, as in many EU countries, hinged to a great extent on reforms of the health sector (6–8). Thus, a series of measures were implemented, including, among others, health workforce downsizing, the reduction in fees paid to health providers, the lowering of pharmaceutical prices, and the setting of pharmaceutical budget equal to a fixed share of the gross domestic product (GDP) (as presented in paragraph 2.1.1.2).

The impact of many of these interventions on the real Greek economy remains unexplored, forming a novel field of applied fiscal statistics which the present work attempts to deal with. It focuses on a pharmaceutical policy intervention with tangible accounting outcomes and *de jure* dependence on economic growth indices (as explained in the following paragraphs), aiming to address a particular angle of the sensitive topic of restrictive healthcare policies’ financial effects on the real economy’s flows.

## Materials and Methods

### Problem formulation: Conceptual framework

#### Curtailing Public Pharmaceutical Expenditure in Greece: A Fiscal Consolidation Strategy

##### Urgency of measures

**Figure 1:**
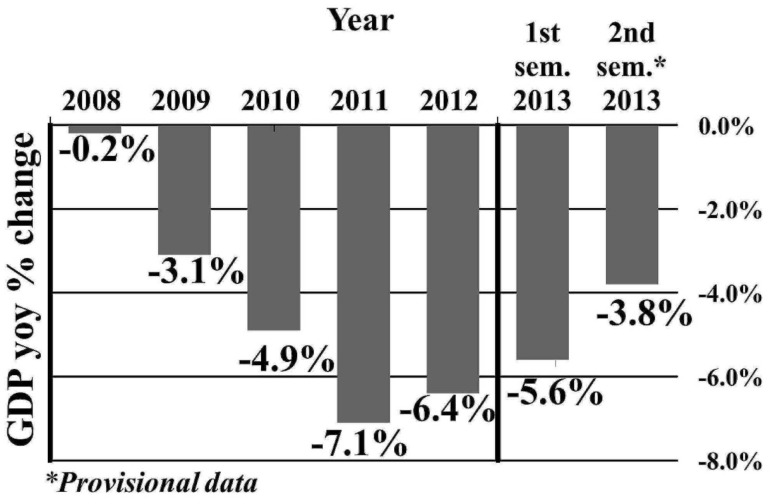
**GDP year-on-year changes in Greece (2008–2013)**.

Upon the advent of the financial crisis in 2008, Greece was already placed at a disadvantaged financial situation due to its high general government gross debt, the highest among EU countries^1^. From 2008 to 2013, the Greek economy suffered a sharp shrinkage: the GDP with a year-on-year fall of up to 7% (Figure 1) fell by 22% over the same period (in per capita terms)^2^ and about one-fifth of the aggregate production was lost [reaching even 2/3 in specific economy sectors, such as the Structure (9)]. Private investments and consumption were reduced by €27.4 and €16.3 bn (or 17.6 and 9.1%), respectively (9), whereas unemployment climbed to 27.4% (first quarter of 2013) from 7.6%, affecting mostly young people and women. The prolonged election period (May–June 2012) deteriorated further the business expectations and consumers’ confidence (10, 11).

In Greece, small deviations from the bottom of the recession were observed from the fourth quarter of 2012 onward, driven mostly by the negotiation with the European Commission (EC), the European Central Bank (ECB) and the International Monetary Fund (IMF), the materialization of commitments on the basis of the Memorandum of Understanding (MoU) and the Memorandum of Economic and Financial Policies, and the withdrawal of €34.4 bn from the support mechanism (10).

The fiscal consolidation strategy of 2014 entailed the application of measures for debt reduction to 174.8%, for primary surplus 1.6%, and for growth 0.6% of the GDP^3^.

**Figure 2:**
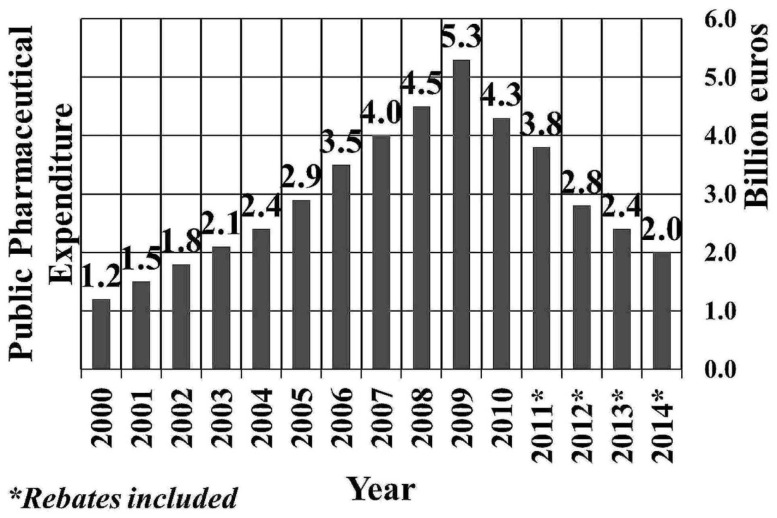
**Public pharmaceutical expenditure of Greece (2000–2014)**.

All economies deal with finite resources, the allocation of which determines the selection among competing strategies and interventions (12). However in the case of Greece, under the IMF loan conditions (13) and according to the MoU, specific economic sectors, such as healthcare, were identified early on, as fields of immediate intervention for fiscal consolidation (14, 15). Public pharmaceutical expenditure (PPE) in particular, having exhibited a sharp expansion in the pre-crisis period (Figure 2), became the capping stone of the country’s effort for a rapid cost-containment in healthcare.

##### Regulatory framework

Since 2010, a significant number of measures have been implemented for the restriction of excess public pharmaceutical expenditure which stemmed from the over-prescription of branded drugs (16). These measures focused on the demand (e.g., generics prescribing, development of therapeutic protocols, etc.) or the supply side (e.g., reimbursement lists for pharmaceuticals, external reference pricing, etc.) of the pharmaceutical market (17).

In 2012, a mechanism referred to as *clawback*, for the automatic refund of amounts that excess the state’s public pharmaceutical expenditure budget is instituted by law (laws 4052 and 4093, g.g. A41 and A222, respectively^4^). Following a ministerial decree (g.g. B2243/2014^4^), the ceiling for pubic pharmaceutical expenditure is specified at 1% of the country’s GDP, which is applicable as from 2014.

##### Accounting and employment outcomes

Over the years 2009–2013, public pharmaceutical expenditure has been reduced by €2.7 bn (or 50%) (Figure 2), which corresponds to 11% of the total amount of reduction of aggregate public expenditure (that is, €24.9 bn) (18). At the same time, according to the Vocational Insurance Fund of pharmaceutical corporations’ employees, the workforce of the medicine branch (corporations and pharmacies) was reduced from 31,100 to 21,500, that is, by 30.9%, yielding a loss of 3.5 job positions for each million cut [i.e., (21,500–31,100)/2,740].

#### Contribution of the Pharmaceutical Branch to the Greek Economy

The present section draws on published data of Greek organizations offering an overview of the pharmaceutical sector^5^.

Public pharmaceutical expenditure (PPE) is a basic component of the country’s economy. In per capita terms, it consists around 44% of the public health expenditure^6^ and 1.9% of GDP (2011) (19).

Considering production value, extroversion, and impact on other sectors of the economy (externalities), the pharmaceutical sector, represents one of the most dynamic sectors of the Greek economy, including 421 pharmaceutical marketing authorization holders, 124 pharmaceutical wholesalers, and 11,315 pharmacies.

In terms of gross value added, the pharmaceutical sector has followed an upward trend in the past decade, reaching 9.6% of total manufacturing production, which represents one of the highest rates in the EU, after Slovenia (12.7%) and Denmark (10.4%). Indeed, during the period 2000–2010, the industry not only increased the percentage of manufacturing production in Greece, but also presented the highest average annual increase in the gross value added among EU countries (by about 7%, from 2.7% in 2000 to 9.6% in 2010). In 2010, the production and marketing of pharmaceutical products contributed to the Greek economy by €7.5 bn which corresponds to 3.4% of the GDP. Furthermore, the industry has invested significant amounts on research and development over time (for instance, €85 and €88 million in 2011 and 2012, respectively).

In terms of employment, only within a year (2010), 132,787 jobs were offered, while total tax revenues from the pharmaceutical sector amounted to €400.9 million. Exports of pharmaceutical products had the fourth largest share in total manufacture exports (6% on average over the years 2000–2010), with the largest percentage of exports being directed to the EU member states. High levels of demand have also been generated on behalf of South Africa, Turkey, Switzerland, and Brazil.

Another area of contribution of the pharmaceutical sector to the Greek economy is that of public debt management, through the settlement of outstanding debts via the bond market and the maturity of public obligations toward pharmaceutical companies (law 4046, g.g. A28/2012 and article 12 of law 4052, g.g. A41/2012 as revised by law 4093, g.g. A222/2012^7^).

The pharmaceutical sector has additionally exerted a considerable impact on the function of other sectors, such as Advertising, which in recent years has suffered a sharp decline of its total turnover. Total advertising costs of the pharmaceutical sector are estimated at €35–€41 million (data on 2012 and 2011, respectively).

### Scope

#### Objectives

Considering the economy’s ongoing shrinkage due to the economic crisis, the issue of applying a health measure that depends on the GDP is challenging. In this respect, the present study attempts to assess the impact of setting public pharmaceutical economy equal to a small share of the (gradually diminishing) GDP on the Greek economy.

In particular, the impact is assessed in terms of the losses in taxes and social security contributions that derive from the reduction of turnover and the increase of unemployment in the pharmaceutical industry and pharmacies. It is worth noting herein that only the direct budgetary impact of reducing public pharmaceutical expenditure is investigated, without considering the potential impact on other sectors or economic consequences, such as the problematic access to treatments due to the ongoing shrinkage of the market (20) and the delays in reimbursement for retail pharmacies (21) as well as the consequent productivity losses related to increase in the length of hospitalization (22, 23) and job absenteeism (24).

#### Working Assumption

In the context of the present analysis we made the working assumption that as pharmaceutical expenditure decreases, the (negative) impact on public revenue is expected to grow, because, in the early years of the measure’s application, the decreases will primarily relate to waste and unnecessary spending.

### Quantitative analysis

We took into consideration the vulnerability of two categories of economic flows. The first corresponds to the annual public cost that is generated by the provision of pharmaceutical care in Greece. The second refers to the net worth resulting from tax collection as dominant share of revenue for government (1), as well as the compulsory transfer of social security contributions.

We developed a calculus for measuring the lost governmental revenues due to the reduction in private and corporate tax amounts and social security contributions [formulas (1)–(1.3)]. Only the direct budgetary impact of the measure is investigated, not taking into consideration the putative impact on other production sectors, as specified earlier.

For the estimation of the effect of the fiscal policy under analysis (namely the PPE curtailment to 1% of the GDP) on the economy, multipliers are used as common quantitative tools. The multiplier is computed dividing cumulative losses of revenues by the observed reduction in the PPE in the time domain 2009–2013 [formula (2)].

We also perform sensitivity analysis for the values of the multipliers. Thresholds of the multiplier are derived by simply leaving indirect taxes (VAT amounts) and unemployment benefits out of the total loss equation [formula (1)].

Finally, three naïve estimating models for the multipliers are fitted to the data, a linear (Eq. 3), an exponential growth (Eq. 4) and a quadratic trend (Eq. 5). Projections on 2015 are performed, using the statistical software Minitab Release 14. The following relationships hold.

(1) $$\text{TR}{\text{L}}_{{\text{t}}_{\text{n}}}\equiv \sum_{\text{i}=1}^{\text{n}}\text{R}{\text{L}}_{{\text{t}}_{\text{i}}}=\text{dP}{\text{R}}_{\text{n}}+\sum_{\text{i}=1}^{\text{n}}\text{U}{\text{B}}_{{\text{t}}_{\text{i}}}$$

(1.1) $$\text{dP}{\text{R}}_{\text{n}}=\text{P}{\text{R}}_{{\text{t}}_{\text{0}}}-\text{P}{\text{R}}_{{\text{t}}_{\text{n}}}$$

where ${\text{TRL}}_{{\text{t}}_{\text{n}}}$ is the total losses in public revenue between years t_0_ and t_n_, with t_n_–t_0_ = n, ${\text{RL}}_{{\text{t}}_{\text{i}}}$ is the loss of government revenue in the year t_i_, i = 1, 2, …, n from the previous year, ${\text{UB}}_{{\text{t}}_{\text{i}}}$ is the unemployment benefits in year t_i_, i = 1, 2, …, n, and PR_k_ is the revenue in the t_k_ year, k = 0, 1, 2, …, n, defined as follows:

(1.2) PR=DT+VATL+SSC

(1.3) $$\text{DT}=\text{D}{\text{T}}_{1}+\text{D}{\text{T}}_{\text{2}}$$

where DT is the direct taxes, DT_1_ is the losses in personal income taxes in year t, DT_2_ is the losses in corporate income taxes in year t, VATL is the value added tax losses, i.e., losses in indirect taxes in year t, and SSC is the social security contributions.

(2) $${\text{m}}_{\text{t}}=\frac{\text{TR}{\text{L}}_{\text{t}}}{\text{dPP}{\text{E}}_{\text{1}}}$$

where m_t_ is the value of the multiplier in the year t, TRL_t_ is the total estimated revenues loss in the year t, and dPPE_1_ is the size of annual cutting down on PPE between two consecutive years.

(3) $${\hat{m}}_{t}=\text{a + b}*\text{t}$$

(4) $${\hat{m}}_{t}=\text{c}*{\text{d}}^{\text{t}}$$

(5) $${\hat{m}}_{t}=\text{e}+\text{f}*\text{t + g}*{\text{t}}^{\text{2}}$$

where ${\hat{m}}_{t}$ is the estimated value of the multiplier in the year t, and a, b, c, d, e, f, g coefficients with a, b, c, e, f, g ∈ R, $d\in R_{+}^{*}$.

## Results

### Total losses

Table 1 presents the estimated annual losses in public revenues that are caused by the downsizing of the pharmaceutical market.

**Table 1:** **Losses per year in government revenues due to the public pharmaceutical expenditure curtailment (2009–2013)**.

Revenue loss category	Absolute change (in € million)				
	2010–2009	2011–2010	2012–2011	2013–2012	2013–2009
Direct taxes (DT)	73.78	37.68	77.15	75.37	263.98
Indirect taxes (VATL)	2.50	5.27	4.26	17.75	29.79
Social security contributions (SSC)	9.50	20.05	16.21	67.55	113.31
Subtotal-RL	85.78	63.00	97.63	160.67	407.08
Unemployment benefits (UB)	9.58	0.48	8.14	27.78	45.98
Total-TRL	95.36	63.48	105.77	188.45	453.06

Total losses of revenues equal €453 million. Nearly half (41.6%) of these losses are observed in the period 2012–2013.

Since dPPE_2013_ equals €400 million (Figure 2) and TRL_2013_ equals €188.5 million (Table 1), the multiplier is estimated at 0.47 for the year 2013. Based on this estimation, €100 million of PPE curtailments are expected to generate losses of €47 million in public revenues.

**Figure 3:**
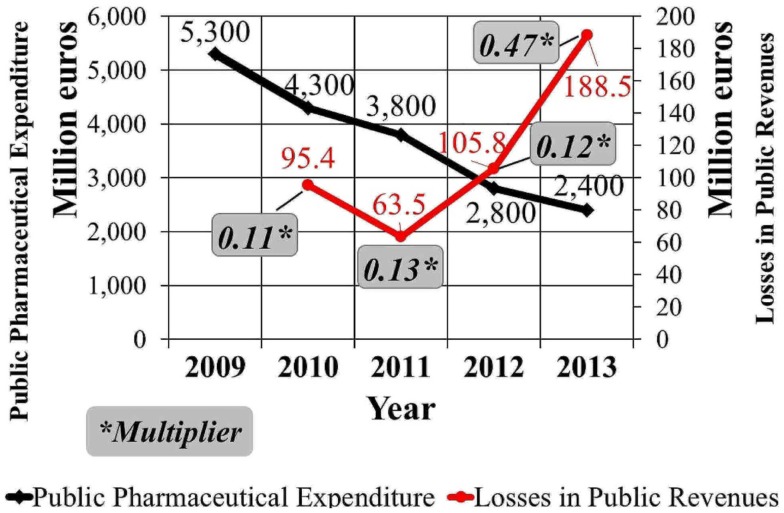
**Public pharmaceutical expenditure changes in Greece and the multiplier (2009–2013)**.

Figure 3 depicts estimations of the multiplier for the period 2009–2013 and combines it with information on public pharmaceutical expenditure and losses in public revenues for the same period.

### Sensitivity analysis

The derived threshold for the estimated multiplier is 0.35 in 2013 meaning that for every €100 million of reduction in the PPE, the losses in public revenues are anticipated to be €35 million at least.

### Projections

**Figure 4:**
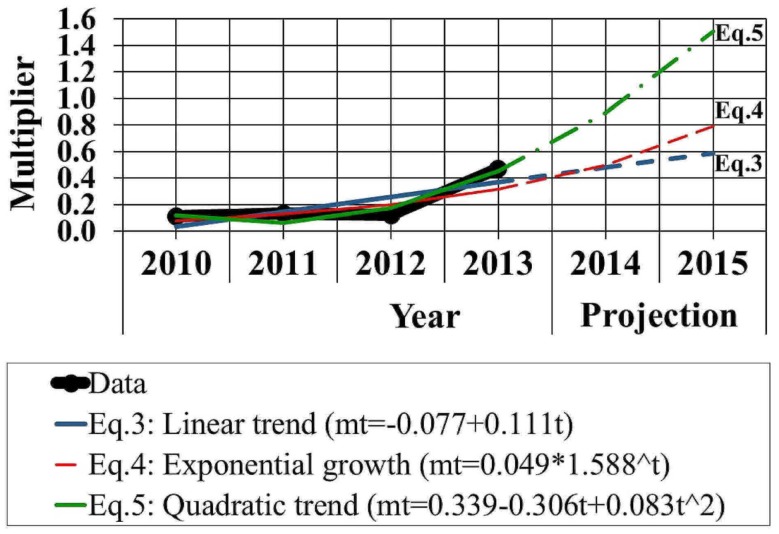
**Projection of the multiplier based on linear trend, exponential growth curve, and quadratic trend models**.

Results based on Eqs 3–5 are depicted in Figure 4. According to the linear and exponential growth trends, the multiplier exceeds 0.5 in 2015, which implies the counterbalance of PPE curtailment by 50% at least. With the quadratic model, the multiplier reaches and exceeds the value 1.0 in 2014 and 2015, respectively.

## Discussion

### Contribution of the analysis

Analysts in Greece have substantiated the pharmaceutical sector’s contribution to the Greek economy on the basis of its high partial multipliers on GDP (2.43), gross value added (2.56), employment (2.48), as well as tax revenues (1.55), compared to other sectors, such as tourism and shipping^8^. In the context of this analysis, we strengthen the existing theoretical framework focusing on an intervention which was adopted as a suppressive measure of excess public pharmaceutical spending. Instead of studying the gains earned from the pharmaceutical sector, we reverse the problem, by assessing the losses stemming from its exhaustion, which to the notion of the authors is an original approach in the field of pharmaceutical policy.

Analytically, this study deployed a simplistic multiplier-based tool for evaluating the impact of a fiscal measure in the time domain 2010–2013 and further, for estimating its dynamics in the near future. This period is short, yet it isolates the crucial historical (i.e., political and institutional) events that are instrumental for the evolution of public pharmaceutical expenditure, namely the MoU and the country’s major healthcare reform (that established electronic prescribing in 2010^9^ and integrated the health branches of social security funds into a single health care provider in 2011^10^).

### Key findings

In the present analysis, we highlighted the weaknesses and short-term risks that accompany the restrictions imposed on the state’s expenditures for pharmaceutical care. Based on the calculated values of the multiplier, a considerable share (35–47%) of the outcome of pharmaceutical spending cutback is expected to be counterbalanced by the yielded losses in public revenues (taxes and contributions). The multiplier increases sharply to 0.47 in 2013, complying with the working assumption that the application of a restrictive pharmaceutical policy is expected to have a bounded period of efficiency, after which further reductions in PPE shall have repercussions on public revenues and the economy as a whole.

The projection procedure indicated that the effects of the applied measure on the GDP are sizeable enough to make the intervention obsolete in the near future. The linear and the exponential growth approach yielded more conservative and similar results. On the contrary, the quadratic trend model generated a sharp projection beyond the value “1,” from 2014 onward. This outcome could be thought of as extreme, yet it is compatible with similar assessments from the literature, regarding government spending multipliers’ propensity to increase, reach or exceed the value “1,” under certain circumstances, in economic downturns (25).

### Conclusion

The pharmaceutical market, an already regulated sector due to the existing external reference pricing system (26), is important for yielding net worth in the Greek economy. Unambiguously, pharmaceutical policies have so far enabled the rationalization of public health expenditures. However, the activation of the clawback mechanism as explained herewith has important side effects, such as the increase of unemployment and the reduction in main sources of public revenue, which undermine the values of solidarity in financing, equity of access, and the provision of high-quality health care (27).

### Prospects of further analysis

The compilation of healthcare data and the subsequent broadening of time series will allow further development of estimation and forecasting tools. In this context, applying multiplier-based analyses to other areas of health policy intervention is considered a reliable methodological vehicle for taking policy-makers a step forward in evidence-based healthcare decision making.

## Conflict of Interest Statement

The authors declare that the research was conducted in the absence of any commercial or financial relationships that could be construed as a potential conflict of interest.

## Footnotes

^1^Eurostat. General government gross debt – annual data. Available from: http://ec.europa.eu/eurostat/tgm/refreshTableAction.do?tab=table&plugin=1&pcode=teina225&language=en. Accessed 22 February 2015.

^2^Based on the authors’ calculations. Source of data: Eurostat. Available from: http://ec.europa.eu/eurostat/tgm/table.do?tab=table&init=1&language=en&pcode=tec00114&plugin=1. Accessed 16 June 2015.

^3^Source: Ministry of Finance, State Budget 2014. Available from: http://www.minfin.gr/?q=el/content/%CE%BA%CF%81%CE%B1%CF%84%CE%B9%CE%BA%CF%8C%CF%82-%CF%80%CF%81%CE%BF%CF%8B%CF%80%CE%BF%CE%BB%CE%BF%CE%B3%CE%B9%CF%83%CE%BC%CF%8C%CF%82-2014-0

^4^Source: http://www.et.gr. Accessed 16 June 2015.

^5^Institute of Social and Preventive Medicine of Greece (IKPI). The impact of Pharmaceutical Expenditure Reduction on Public Revenues Athens, April 2014

^6^Calculation of the authors. Source: http://dx.doi.org/10.1787/health-data-en; WHO Global Health Expenditure Database data-en.

^7^Source: http://www.et.gr. Accessed 12 April 2015.

^8^Foundation for Economic and Industrial Research (IOBE). Economic footprint of production and distribution of pharmaceutical products in the Greek economy. Athens, January 2013.

^9^Law 3982 “Electronic recording and processing of prescriptions and medical examinations,” g.g. 189/2010. Available from: http://www.et.gr. Accessed 10 June 2015.

^10^National Organisation for Health Care Services Provision (EOPYY). Law 3918 “Structural reforms of the health system and other provisions,” g.g. A31/2011. Available from: http://www.et.gr. Accessed 10 June 2015.

## Abbreviations

DT, direct taxes; PPE, public pharmaceutical expenditure; PR, revenue; RL, revenue losses, SSC, social security contributions; TRL, total revenue losses; UB, unemployment benefits; VATL, value added tax losses.
